# Research Capacity and Training Needs for Cancer in Conflict-Affected MENA Countries

**DOI:** 10.5334/aogh.2809

**Published:** 2020-11-06

**Authors:** Zahi Abdul-Sater, Elsa Kobeissi, Marilyne Menassa, Talar Telvizian, Deborah Mukherji

**Affiliations:** 1 Global Health Institute, American University of Beirut, Beirut, LB; 2 Naef K Basile Cancer Institute, Faculty of Medicine, American University of Beirut, Beirut, LB

## Abstract

**Background:**

The global cancer burden is disproportionately greater in low- and middle-income countries, including those affected by conflict in the Middle East and North Africa (MENA) region. Contributing factors include inadequate control of risk factors plus limited surveillance and treatment options. Weak healthcare infrastructure may be further compounded by the conflict prevalent in multiple MENA countries. Improved cancer surveillance, research, and capacity strengthening are essential for implementing cancer control plans in the MENA region, requisite for reducing the disproportionate cancer burden.

**Aims:**

This article aims to understand the barriers to cancer research and training in conflict-affected MENA countries, and to identify opportunities for developing capacities for reliable cancer research strategies.

**Methods:**

This study employs a mixed-method approach utilizing an online questionnaire with open and close ended questions targeting oncologists and cancer researchers in conflict-affected MENA countries. For open-ended questions, we performed a qualitative content analysis to identify thematic barriers.

**Results:**

Forty-eight respondents, mostly Medical and Radiation Oncologists, completed the questionnaire. The most significant training needs were conducting clinical, basic, and qualitative cancer research. The most prominent barriers identified were insufficient training in data analysis and research design (77% and 75% of respondents, respectively) and insufficient institutional and government funding (94% and 85%, respectively). For the qualitative data, we organized the barriers into six themes, the most common was the lack of research infrastructure (28%).

**Conclusions:**

Despite an escalating cancer burden, conflict-affected MENA countries are lagging in knowledge production and implementation of evidence-based cancer research. Novel modes of knowledge transmission and collaboration across geographical and political boundaries are sorely needed. Based on our study, we recommend developing innovative and accessible training opportunities focusing on developing basic, clinical, and qualitative research skills. Research capacity-strengthening initiatives should encourage the investigation of context-specific research questions with the potential to make a meaningful impact on cancer control in the region.

## 1. Introduction

The global burden of cancer is disproportionately greater in low- and middle-income countries (LMICs), including those affected by conflict in the Middle East and North Africa(MENA) region [1]. LMICs shoulder more than 50% of the 14.1 million cancer cases worldwide in addition to a projected 60% increase in the cancer burden by 2030 [2]. This burden is expected to continue shifting towards LMICs as the global population grows and ages in addition to the massive epidemiological transition to non-communicable diseases in LMICs [3,4,5]. The underlying factors contributing to this disparity include limited availability and accessibility to surveillance, screening and treatment, inadequate control of risk factors, and weak healthcare infrastructures [6]. These challenges are further compounded by the fragmentation of healthcare caused by the protracted conflicts prevalent in multiple MENA countries.

Cancer surveillance, research and capacity building are essential for establishing national and regional cancer control plans in the MENA region, requisite for reducing the disproportionate cancer burden [7,8]. The World Health Organization (WHO) emphasizes that capacity building should extend beyond building infrastructure and resources to strengthening capacity for high-quality research within the country [9]. Nevertheless, the capacity for conducting health research, including cancer research, in the MENA region is limited [10]. This weakened capacity has been attributed to fewer researchers, weak research infrastructure, underdeveloped cancer registries, limited collaboration within the scientific community, low spending on research, and cultural barriers affecting patient recruitment in clinical studies. Moreover, recurrent conflicts and political unrest that plagues the region often result in migration of researchers and reduction in funding, which further reduces the capacity for conducting research [7,10,11,12,13] Consequently, knowledge production in the field of oncology is compromised in the MENA. A recent study examining the trends in cancer research in the Arab world highlights that knowledge production in oncology is limited in both quantity and quality. In addition to the low number of publications in comparison with the USA and Japan for instance, most publications were case reports and descriptive in design. A dearth of high-quality studies was observed, including cross-sectional, randomized controlled trials (RCTs) and systematic reviews/meta-analyses [7,14].

Strengthening capacity for cancer research in the MENA is essential in devising contextualized strategies to improve treatment across the cancer continuum (prevention, early detection, diagnosis, treatment, and palliation) [11,15]. Furthermore, strengthening cancer research will help build the evidence base needed not only to reduce cancer incidence, mortality, and morbidity but also to plan proper policies for better cancer control [16]. Strengthening capacity for cancer research requires developing training that accounts for the specific training needs and multifactorial barriers experienced by the cancer researchers and oncologists in the MENA countries affected by conflict. Against this backdrop, this article aims to understand the barriers to cancer research in conflict-affect countries in the MENA region, allowing for a deeper understanding of the context in which cancer research is hindered and for identifying opportunities for developing reliable cancer research strengthening strategies. The article also identifies training recommendations and needs of oncologists in areas of conflict, which would help inform future capacity strengthening activities for research on cancer.

## 2. Methods

This study is a part of the R4HC-MENA project funded by the United Kingdom Global Challenges Research Fund (GCRF). The aim is to build research capacity and policy in conflict-affected areas focusing on non-communicable diseases, including cancer. This study employs a mixed-method approach utilizing a self-administered online survey. The survey includes the Hennessy-Hicks Training Needs Analysis Questionnaire (HH), a validated training requirement evaluation tool [17]. The HH questionnaire content was adapted to the needs of the study and, consequently, cannot be compared with other needs assessments using the same questionnaire.

### 2.1. Survey Development

The survey comprises 24 questions relating to general information on respondents (8 questions), training methods of delivery (6 questions), perceived barriers to research capacity and potential training topics (3 questions), training needs assessment (1 question), and barriers to conducting cancer research in conflict (6 questions).

The training needs assessment included 21 items. Each item was rated along a 7-point scale for how important the activity is to the respondent’s job (A) is and how well the respondent performs the activity (B). The difference in scores (A vs. B) provides an assessment of where the greatest needs lie.

### 2.2. Survey Population

The target population for the survey was oncologists and cancer researchers in conflict-affected MENA countries. To identify conflict-affected countries, we selected the countries that exist in both the World Bank list of MENA countries and fragile and conflict-affected settings. The resulting list included the following countries: Djibouti, Iraq, Lebanon, Libya, Somalia, Sudan, Syrian Arab Republic, Yemen, and Palestine.

To find respondents from the countries mentioned above, we extracted e-mails from online medical directories and academic publications originating from those countries. We also employed snow-ball sampling to help increase the number of respondents. We were not able to identify potential respondents from Somalia and Djibouti.

### 2.3. Administration

After institutional review board (IRB) approval, we used the web-based tool, LimeSurvey, to collect survey data from the convenience sample previously described. The survey was administered in Arabic and English after being pilot tested by four medical residents at the American University of Beirut Medical Center (AUBMC). Overall, the pilot testers described the survey as concise. We made minor modifications based on their feedback, including changing terminologies and defining unclear concepts.

E-mails were sent to 280 individuals, of which 35 bounced back as undeliverable. The e-mails were sent in both English and Arabic and explained that the survey aims to assess the current needs and challenges of cancer research in conflict-affected MENA countries. Reminder e-mails were sent two and four weeks after the first email. A separate e-mail was sent to those who completed the survey with a request to electronically disseminate the questionnaire to their colleagues who work in cancer care or research. A total of 48 respondents completed the survey.

### 2.4. Analysis

Survey responses were exported into Microsoft Excel, where the data was managed and cleaned. For the Training Needs Assessment (TNA), a parametric T-test, using GraphPad, was employed to explore the statistically significant difference between score A (importance of activity to job) and score B (ability of respondent to perform the activity), as recommended by the HH tool authors [17,18]. For open-ended questions, we performed a qualitative content analysis to identify thematic barriers for cancer research and for attending training. Two researchers (ZAS and EK) independently read and coded the open-ended responses into themes and sub-themes concerning the barriers of cancer research and training. Final themes were agreed upon by discussion with the research team. Examples of responses provided in the manuscript were edited for clarity and conciseness. Original responses can be found in (supplemental file).

## 3. Results

### 3.1. Participants Profile

**Figure 1:**
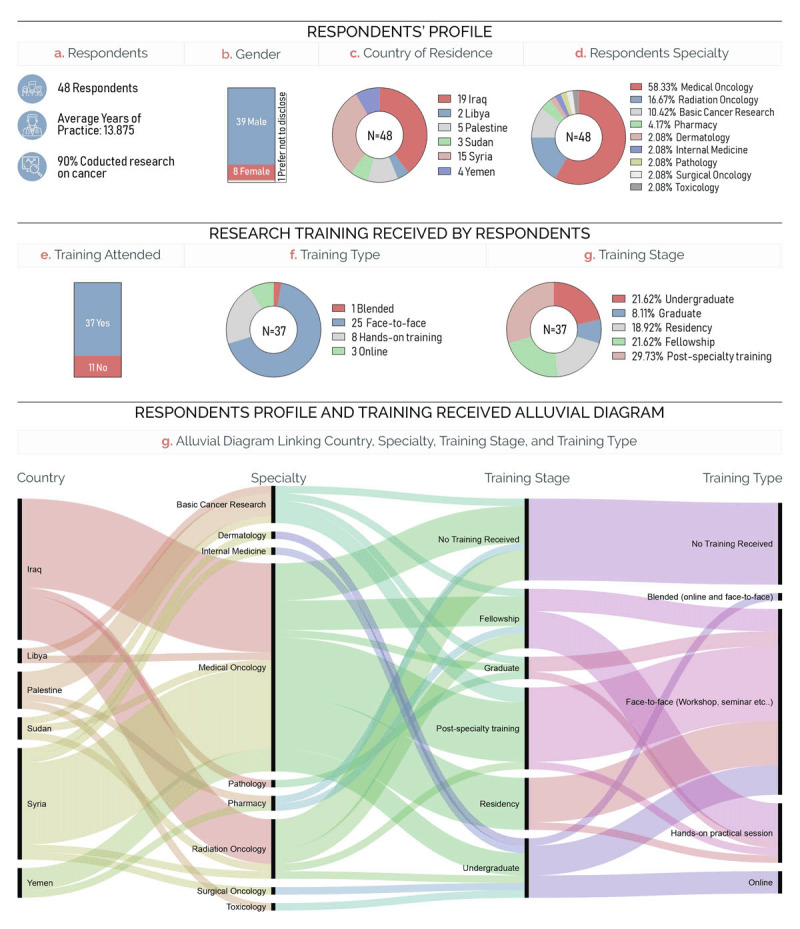
Demographics **(a–d)** of and training received **(e–g)** by survey respondents. **(h)** Alluvial diagram connecting the respondents’ country, specialty, training stage, and training type.

**Figure 2:**
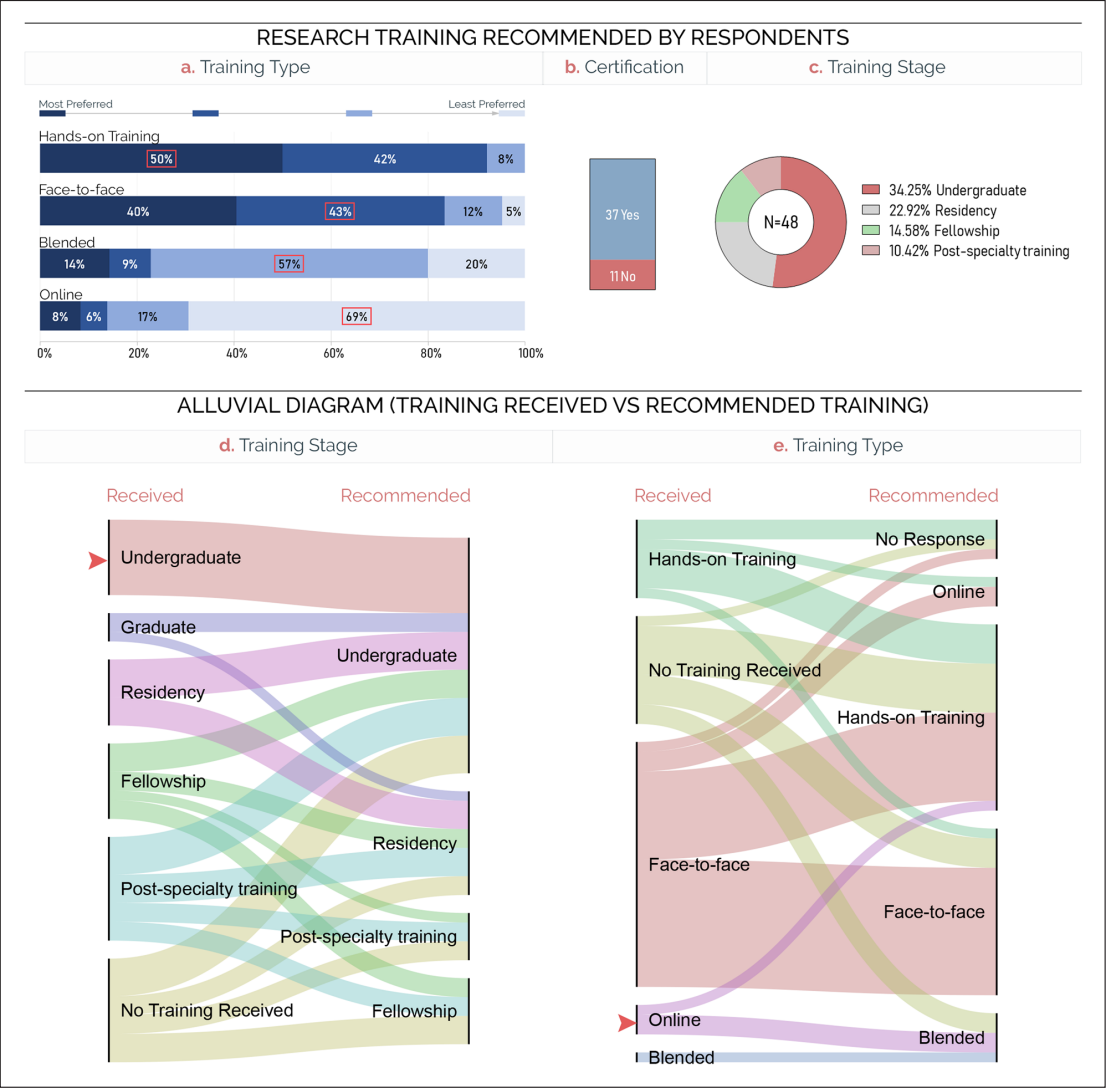
Training type **(a)**, certification **(b)**, and training stage **(c)** as recommended by survey participants. Alluvial diagram showing the relationship between training stage **(d)** and type **(e)** received and recommended by survey participants.

A total of 48 respondents (17.1%) completed the questionnaire, out of which 39 were males and 9 were females (Figure 1a and b). Most respondents are Medical and Radiation Oncologists (75%) that have conducted research on cancer throughout their career (90%) (Figure 1a and d). With an average of 13.9 years of practice, they mostly originate from Iraq and Syria, in addition to Palestine, Sudan, Libya, and Yemen (Figure 1a and c). Thirty-seven respondents received at least eight hours of research training, mostly face-to-face (25 respondents) and hand-on (eight respondents) sessions at the undergraduate (26.62%), graduate (8.11%), residency (18.92%), fellowship (21.62%), and post-specialty (29.73%) levels (Figure 1f and g). Interestingly, the alluvial diagram highlights the eclectic nature of the respondents’ specialties regarding their country of origin and points out that all the online training attended were at the undergraduate level (Figure 2). The demographics of the survey participants reveal that they are well-experienced practitioners that have attended training on cancer and cancer research.

### 3.2. Training Recommendations

Developing research training that is adapted to the contextual needs and recommendations of oncologists and cancer researchers is necessary to ensure its success and efficacy. Survey participants were asked to give their recommendations for the training type, stage, and certification (Figure 2). Hand-on or face-to-face training at the undergraduate or residency level that leads to certification was the most recommended training formula (Figure 2a–c). Interestingly, all respondents that received research training at the undergraduate level recommended training at the same level (Figure 2d). Furthermore, online training was the least preferred and was not recommended by respondents who attended online training during their careers (Figure 2e).

### 3.3. Training Needs Assessment

**Figure 3:**
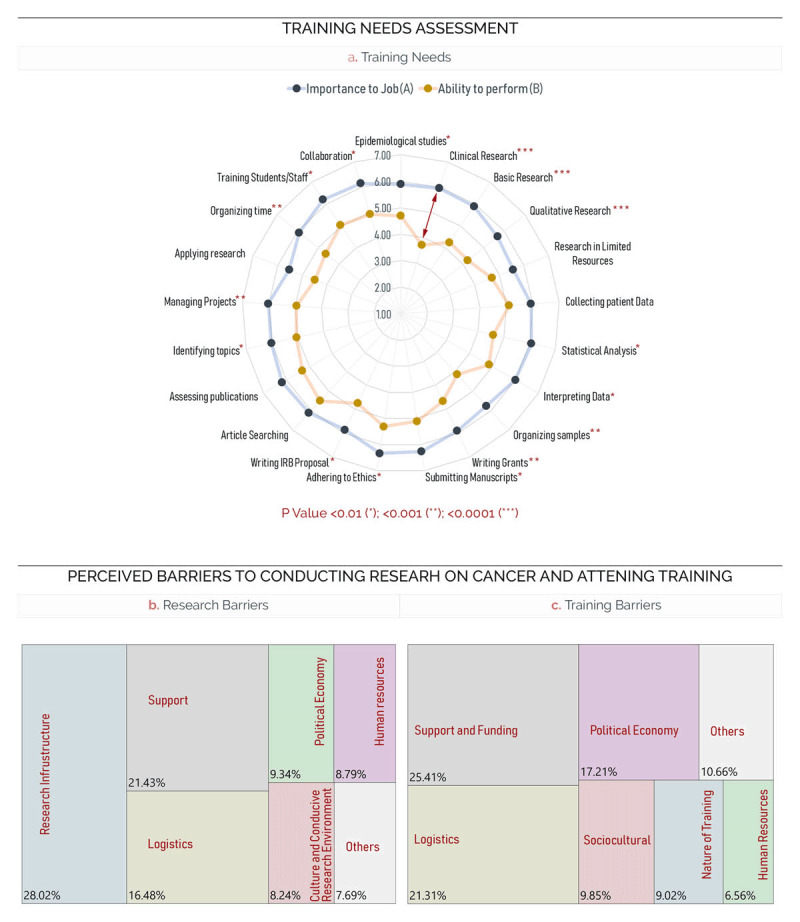
**(a)** Radar chart highlighting the difference between the importance to job and ability to perform in regard to activities related to cancer research and care. Treemap chart showing the themes mapped from participants’ responses to the research **(b)** and training **(c)** barriers.

Next, we asked participants to rate 21 activities related to cancer research and care in terms of importance to the job (Score A) and ability to perform (Score B), on a scale from 1 to 7. Comparing the difference between the means of scores A and B for each activity, we identified significant training needs across the participants (Figure 3a). The most significant training needs (p < 0.0001) were conducting clinical research on cancer (e.g., clinical trials); conducting basic cancer research; and conducting qualitative research in cancer. These data highlight the great need for strengthening capacity for cancer research methodologies. Other significant training needs (p < 0.001) pertained to the management of research and practice and included activities like properly organizing and storing tissues/samples; writing a grant proposal; designing, supervising, and managing cancer research projects; and organizing your own time effectively in a conflict setting. Other training needs are shown in Figure 3a and supplemental Table 1.

### 3.4. Perceived Research and Training Barriers

Next, we sought to understand the perceived barriers to cancer research in conflict settings. We probed the participants to select preset barriers on the individual (supplemental Table 2), institutional (supplemental Table 3), linkages and collaboration (supplemental Table 4), organizational system and enabling environment (supplemental Table 5), and political economy levels (supplemental Table 6). The most prominent individual barriers were insufficient training in data analysis and research design that were selected by 77% and 75% of respondents, respectively. These results mirror the findings in the Training Needs Assessment (TNA) discussed in the previous paragraph and highlight the need for training that targets individual research skills. On the institutional level, insufficient funding (94%) and a lack of multidisciplinary research teams (83%) topped the selected choices by participants. The unanimity of the selection underlines the need for and importance of securing institutional funding to ensure proper research on cancer. On the other hand, insufficient governmental (85%) and non-academic partnerships (81%) were the most selected obstacles on the level of the partnerships and collaborations. We also sought to understand how the environment and organizational system around cancer research might be hindering factors in conflict-affected settings. Eighty-five percent of respondents selected insufficient opportunities for fellowships, scholarships, and/or applications, and 75% selected inadequate protected time for research activities. Lastly, the majority of the participants agreed that insufficient government funding (94%) and policies that support research on cancer (92%) constitute the primary obstacles within the political economy realm.

To gain an unguided, in-depth understanding of the cancer research and training obstacles, the survey participants were invited to list up to five barriers for conducting cancer research and for attending training events (Figure 3b and c). In total, we received 158 research barriers and 114 training barriers. We organized the research barriers into six themes, out of which 15 sub-themes were identified (supplemental Table 7). Overall, out of 158 participants’ responses, we found 168 mentions of one of the six research barriers themes. Twenty-eight percent of the mentions focused on the lack of research infrastructure, including amenities (e.g., facilities and equipment), research resources (e.g., reagents and cancer drugs), data poverty (e.g., availability or quality of cancer data and statistics), and cancer registration. Furthermore, the lack of financial (e.g., lack of funding), institutional (e.g., absence of ethical committees), and governmental support (e.g., lack of governmental funding and support) was heavily mentioned (21%). The third thematic barrier identified was logistics (16%), which included obstacles related to research participation (e.g., patient cooperation), travel (patients unable to reach destination), workload and time, collaboration (e.g., communication between centers), and training (e.g., fellowships). Further, 9% of mentions were mapped into the human resources theme (e.g., absence of mentors) and 8% were mapped into the political economy theme, which included three subthemes: Politics (e.g., absence of MOH role alongside organizational and financial corruption) and security (e.g., risky and unsafe roads). These results highlight the multi-faceted and intertwined nature of the obstacles faced by cancer researchers in conflicted-affected settings within the MENA region.

Next, we mapped the responses from the list of barriers to attending research training into six main themes and twelve sub-themes (supplemental Table 8). Twenty-six percent of the 122 mapped mentions focused on the support theme, which included financial support (e.g., financial limitations and low budget), institutional support (e.g., absence of institutional fellowships and organization), and governmental support (e.g., governments in the Arab world are not interested in organizing training at international universities). Participants also mentioned logistical obstacles (21%) including workload and time (e.g., Busy with private clinical work), travel and visa (e.g., difficulty in obtaining a visa), and infrastructure (e.g., absence of research centers). Politics (e.g., corruption) and security (e.g., risk of mobility) were among the political economy barriers mentioned by participants (17%). Furthermore, 10% of mentions were mapped into socio-cultural barriers like the absence of training and research culture (e.g., underestimation of the value of the research) in addition to lack of incentive to attend training. Interestingly, the nature of the training itself in terms of quality and availability (e.g., lack of hands-on training), and cost (e.g., courses are very pricy) was also considered an obstacle (8%). Lastly, human resources barriers, such as lack of specialized trainers were also listed (7%). These barriers emphasize the need to strengthen capacity for affordable and quality training that overcome the mobility and security barriers.

## 4. Discussion

Despite shouldering a significant proportion of the global cancer burden, countries in the MENA region, especially in conflict-affected settings, are lagging in knowledge production and implementation through evidence-based cancer research. Research barriers include lack of resources, poor access to literature, and inadequate research skills, all of which are further exacerbated in conflict-affected areas [19]. Building research capacity through training that targets the contextual needs of cancer researchers in these areas would facilitate access, dissemination, exchange, and implementation of evidence, ultimately leading to a better understanding of cancer and enhanced cancer care. In this article, we used a survey questionnaire to tease out the training needs and recommendations of cancer researchers and the barriers they face to conduct research and attend training in conflict-affected settings in the MENA region.

Originating from conflict-affected countries like Syria, Iraq, and Yemen, survey respondents were mostly medical and radiation oncologists who received training in cancer research. Face-to-face and hands-on sessions dominated the types of training they received, with online delivery ranking last, highlighting the scarcity of online training in such areas (Figure 1f and g). Respondents were then asked to give their training recommendations in terms of stage, format, and mode of delivery (Figure 2). The training topics were identified through Training Needs Assessment (TNA) for activities related to cancer research and care (Figure 3a and supplemental Table 1). The results revealed that a certified face-to-face or hands-on training at the undergraduate or residency levels that builds basic, clinical, and qualitative research skills was the combination recommended by participants.

Understanding the barriers to conduct research and attend training is essential in designing training that is contextualized to overcoming those obstacles. Thus, we probed the respondents to select research barriers on the “Individual”, Institutional”, “Linkages and Collaboration”, “Organizational System and Enabling Environment Barriers”, and “Political Economy” levels (supplemental Tables 2–6). This was complemented with open-ended questions asking the respondents to list barriers for conducting research and attending training (Figure 3b and c, Online Resource 1). In general, the barriers focused on logistics, research infrastructure, human resources capacity, support and funding, culture, and political economy. Indeed, studies within and outside the region noted that logistical difficulties like insufficient time allotted to research prevent physicians from engaging in research activities [7,20,21]. Further, establishing strong research infrastructure, including data production and cancer registration, is essential for developing national and regional cancer control plans. While the Arab region constitutes 5% of the global population, its share of global research expenditure is only 1%, reflecting the paucity in producing data and research [7]. Furthermore, it is well documented, and further confirmed by our survey respondents, that cancer registries in the MENA are underdeveloped. For example, while Australia’s high-quality incidence covers 100% of its population, only 2% and 5% of African and Asian populations are covered, respectively [4]. Human resources capacity was also identified as an obstacle, which is in line with previous studies reporting a decrease in the number of researches in the Arab world [7,20] and confirms the need for strengthening research skills identified in the TNA conducted in this study. Lack of funding and support, on the institutional and governmental levels, was considered a major barrier for both research and training. Indeed, in 2012, Arabic countries only spent 0.562% of their Gross Domestic Product (GDP) on research and development compared to the world and USA average of 2.01%. The lack of funding and support for cancer research and training also stems from the necessity to focus on other health priorities, like controlling infectious diseases. This is compounded by the lack of research culture within the region, which was also identified as one of the barriers in this study. Participating in research studies was mentioned by survey respondents as a barrier, reflecting previous reports of public fear to participate in clinical trials in the region despite valuing the importance of medical research [22]. Interestingly, political economy barriers, especially those pertaining to conflict, were heavily selected and mentioned by survey respondents. Unfortunately, political unrest and conflicts that plague this region compromise the stability of the health systems in place, research infrastructure, and human resources capacity. Indeed, conflict is an all-encompassing theme that permeates all the barriers mentioned above. Dismantlement of health care systems, reduction in funding, migration of skilled researchers, inaccessibility of training due to roadblocks and violence, lack of research amenities due to embargo and unsafe transportation are among the barriers compounded by conflict. Numerous examples exist in the literature that document the effect of conflict on cancer research. UN sanctions on Iraq’s regime on a range of chemotherapy drugs, diagnostic and treatment equipment hindered cancer research and delivery of cancer care [23].

Research capacity strengthening in the MENA region is still lacking. Collaborative initiatives dedicated for capacity strengthening are a must. The Research for Health in Conflict (R4HC) is an exemplar of a collaboration that aims to strengthen health research, including cancer. As part of its efforts in the region, the R4HC consortium aims to design training and courses to build research skills for the health professionals in the MENA, including cancer researchers and oncologists. Another important initiative, the Center for Research and Education in the Ecology of War (CREEW) fellowship which is housed at the Global Health Institute at the American University of Beirut (GHI-AUB), aims to equip frontline health practitioners working in conflict settings with the necessary skills that would enable them to conduct research into the relationship between health and war. Thematic fellowships include Antimicrobial Resistance (AMR) and will be expanded to other health areas, including cancer. Initiatives like R4HC and CREEW rely heavily on designing contextualized courses which should be informed by the needs of the population of interest, including cancer researchers and oncologists. This study provides an assessment of the training needs and barriers that can serve as the basis of designing courses targeting conflict affected MENA countries.

Importantly, the findings in this study need to be considered in light of certain limitations. First, results from convenience and snowball sampling are of unknown generalizability, and the sample may be limited to those interested in the topic. It is important, however, to understand the sampling and its generalizability in the context of small number of oncologists in the conflict affected areas in the MENA region. In a relatively recent study examining the global oncology workforce, no clinical oncologists were identified in South Sudan and only 60 were identified in Iraq, a country with a population of more than 38 million. Second, most respondents are from Iraq and Syria, which affects the representation of the other countries affected by conflict in the region. Lastly, the low response rate (17.1%) might be explained by lack of internet access or migration of potential respondents due to conflict. Most e-mails were extracted from outdated online directories which might also explain the low response rate.

## 5. Conclusion

To our knowledge, this is the first study aiming to inform the design of training for cancer research in conflict-affected settings through understanding the needs and barriers to conducting research and attending training. Cancer researchers and caregivers recommended the nature of training, showcased their training needs, and identified their perceived barriers to research and training. It is vital to ensure that the recommended nature of training takes into consideration the barriers listed. For example, while face-to-face and hands-on sessions were the preferred mode of delivery, in many cases it would not be feasible given that one of the most prominent barriers is unsafe transportation. Consequently, another approach would be taking advantage of online delivery, which would overcome the transportation barrier when needed, be more accessible, and reach a wider audience. Innovative modes of knowledge transmission and evidence accumulation that cross geographical and political boundaries are much needed in conflict-affected MENA countries [24,25]. These knowledge transmission vessels should accommodate the training needs and transcend the contextual barriers within such areas. Based on the results delineated in this study, first, we recommend developing certified training with an online component and opportunities for research mentorship fostering basic, clinical, and qualitative research skills among cancer researchers in the region. Online training may include asynchronous courses, webinars, on-demand and live sessions, plus access to a mentorship program in collaboration with specialized international research centers. Second, the training offered should be multidisciplinary, focusing not only on epidemiological, clinical, and basic research, but also on qualitative research and proper management of practice and research in conflict settings, emanated from daily practice and focused on population needs. Third, capacity strengthening at the individual level through incorporation of research educational programs in universities, with research production being a graduation requirement may be an essential step in changing the research-disregarding clinician culture. Moreover, training of trainers either locally or through international hands-on fellowship programs may be a basis of a hive of future researchers. Nevertheless, capacity building should not be only limited to individual development but should also include strengthening institutional research capacity. Incentives such as promotion points or increased recognition and external funding opportunities may be motivational enough for researchers at academic institutions to increase knowledge output, however, innovative ways to involve community practitioners should be encouraged. Furthermore, it is important to attain governmental support through a nationwide health-research body, preferably under the banner of ministries of health, that unifies and organizes intra and inter institutional collaboration and knowledge transfer, to implement research-based policies and guidelines. Funding remains to be a major issue that may be overcome through grant applications and collaborations with international organizations and stakeholders. Finally, local, regional and international organizations should coalesce to put pressure on policy makers in the region to increase monetary and logistical support for cancer research, stressing on the importance of regional population-derived data to improve cancer control. Based on the data from this study, we are in the process of developing blended learning opportunities including mentorship components. We aim for these programs to be open to as many participants as possible ensuring equitable access to context-specific capacity strengthening initiatives. Empowering regional cancer researchers with the knowledge and support to formulate and investigate research questions of direct relevance to the population they serve will be the over-arching aim of our collaborative work.

## Additional File

The additional file for this article can be found as follows:

10.5334/aogh.2809.s1 Online Resource 1. Two sheets describing themes and subthemes of barriers to conducting cancer research and attending training. The verbatim answers of participants can be seen in column A. The themes and subthemes can be found in row 2 and 3, respectively. Each response was mapped to one or more subtheme.
