# Supplementary material for: Research Capacity and Training Needs for Cancer in Conflict-Affected MENA Countries
Source: Ann Glob Health. 2020 Nov 6;86(1):142. doi: 10.5334/aogh.2809 (PMC7646279; doi:10.5334/aogh.2809)
Supplement: Online Resource 1. — Two sheets describing themes and subthemes of barriers to conducting cancer research and attending training. The verbatim answers of participants can be seen in column A. The themes and subthemes can be found in row 2 and 3, respectively. Each response was mapped to one or more subtheme. [file agh-86-1-2809-s1.pdf]

Activity	Abbreviated Activity Name	Importance to Job	Ability to perform	Difference	Pvalue	Significance
Conducting descriptive epidemiological studies on cancer	Epidemiological Studies	5.89	4.70	1.20	0.0017	*
Conducting clinical research on cancer, e.g. clinical trials	Clinical Research	5.96	3.72	2.24	<0.0001	***
Conducting basic cancer research	Basic Research	5.91	4.26	1.65	<0.0001	***
Conducting qualitative research in cancer	Qualitative Research	5.67	4.24	1.43	<0.0001	***
Conducting research with limited resources	Research in Limited Resources	5.57	4.70	0.87	0.0287	
Collecting own clinical/patient/surveillance data	Collecting Patient Data	5.93	5.11	0.83	0.0623	
Performing proper statistical analysis of cancer data	Statistical Analysis	6.07	4.59	1.48	0.0019	*
Interpreting your own patient data	Interpreting Data	6.00	4.85	1.15	0.002	*
Properly organizing and storing tissues/samples	Organizing Samples	5.76	4.13	1.62	0.0001	**
Writing a grant proposal	Writing Grants	5.91	4.67	1.24	0.0001	**
Submitting a manuscript to a journal	Submitting Manuscripts	6.27	5.11	1.16	0.0035	*
Adhering to ethical guidelines & oversight when publishing	Adhering to Ethics	6.33	5.31	1.02	0.005	*
Writing an Institutional Review Board (IRB) proposal	Writing IRB Proposal	5.87	4.76	1.11	0.0022	*
Using efficient methods of searching articles on the	Article	6.11	5.49	0.62	0.0272	

internet	Searching					
Accessing relevant literature for clinical work	Assessing Publications	6.18	5.30	0.89	0.0179	
Identifying viable research topics in cancer	Identifying Topics	6.02	5.04	0.98	0.0071	*
Designing, supervising, managing cancer research projects	Managing Projects	6.02	4.96	1.07	0.0002	**
Applying research results to your own practice	Applying Research	5.53	4.49	1.04	0.0055	*
Organizing your own time effectively in conflict setting	Organizing Time	5.91	4.63	1.28	0.0003	**
Instructing/training students or junior staff on research in cancer	Training Students/Staff	6.22	5.04	1.17	0.0055	*
Collaborating with other institutions on cancer research	Collaboration	6.15	4.93	1.22	0.0083	*

Supplemental Table 1. Training Needs Assessment (TNA) showing the cancer research and practice activities assessed for their importance to job and ability to perform. The difference between the two and its statistical significance (p-value) is also shown. Abbreviated activity names were used in Figure 5. *** p-value<0.0001; ** p-value<0.001; * p value<0.01

Individual Barriers	Yes	No	Yes (%)	No (%)
Insufficient training in data analysis	37	11	77.08%	22.92%
Insufficient training in research design	36	12	75.00%	25.00%
Not enough incentive to conduct research	33	15	68.75%	31.25%
Inability to identify local or international collaborators	32	16	66.67%	33.33%
Insufficient training in submitting and administering research applications	28	20	58.33%	41.67%
Not enough personal interest in research	18	30	37.50%	62.50%

Supplemental Table 2. Present individual barriers that were selected by participants, shown as raw numbers and percentages.

Institutional Barriers	Yes	No	Yes (%)	No (%)
Insufficient institutional funding for research on cancer	45	3	93.75 %	6.25%
Lack of multidisciplinary research teams	40	8	83.33 %	16.67 %
Unsatisfactory assessment of research needs by the institution	36	12	75.00 %	25.00 %
Research skills are not shared enough among research teams	35	13	72.92 %	27.08 %
Insufficient institutional policies to motivate research among faculty	34	14	70.83 %	29.17 %
Insufficient staff trained in cancer research and research methodology	34	14	70.83 %	29.17 %
Insufficient opportunities for staff research training and professional development	34	14	70.83 %	29.17 %
Inadequate monetary compensation for research staff	34	14	70.83 %	29.17 %
Unsatisfactory facilities and equipment necessary for research	34	14	70.83 %	29.17 %
Insufficient institutional policies that explicitly support and encourage research	31	17	64.58 %	35.42 %
Poorly functioning mentored research program	29	19	60.42 %	39.58 %
Poorly functioning Institutional Review Board (IRB) to review research ethics	27	21	56.25 %	43.75 %
Staff is not held accountable for getting work done according to clear performance standards	26	22	54.17 %	45.83 %
The staff turnover rate is high	15	33	31.25 %	68.75 %

Supplemental Table 3. Present institutional barriers that were selected by participants, shown as raw numbers and percentages.

Linkages and Collaboration Barriers	Yes	No	Yes (%)	No (%)
Insufficient partnerships with government partners	41	7	85.42 %	14.58 %
Insufficient partnerships with non-academic research partners	39	9	81.25 %	18.75 %
Insufficient international collaborations	37	11	77.08 %	22.92 %
Insufficient local collaborations	33	15	68.75 %	31.25 %

Supplemental Table 4. Present linkages and collaboration barriers that were selected by participants, shown as raw numbers and percentages.

Organizational System and Enabling Environment Barriers	Yes	No	Yes (%)	No (%)
Insufficient opportunities for fellowships, scholarships and or applications	41	7	85.42 %	14.58 %
Inadequate protected time for research activities	36	12	75.00 %	25.00 %
Poorly functioning organizational system detailing the role of each team member of a research group	36	12	75.00 %	25.00 %
Poor research culture in the country	36	12	75.00 %	25.00 %
Inadequate research capacity building activities, like workshops and training	34	14	70.83 %	29.17 %
Difficulties in obtaining/organizing data due to conflict	34	14	70.83 %	29.17 %
Inadequate continuing education for clinicians and researchers	32	16	66.67 %	33.33 %
Inability to follow up with subjects due to conflict	32	16	66.67 %	33.33 %
Poorly functioning organizational system for reporting cancer cases	31	17	64.58 %	35.42 %
Poor access to research resources (e.g. time, Internet, Information, equipment)	27	21	56.25 %	43.75 %
Poorly functioning opportunities to present, discuss and publish results	26	22	54.17 %	45.83 %
Health research activities are not relevant to health problems	25	23	52.08 %	47.92 %
Direction of research is dependent on research interests of funders	22	26	45.83 %	54.17 %

Supplemental Table 5. Present organizational system and enabling environment barriers that were selected by participants, shown as raw numbers and percentages.

Political Economy Barriers	Yes	No	Yes (%)	No (%)
Insufficient governmental funding for cancer research	45	3	93.75 %	6.25%
Insufficient government policies that explicitly support research on cancer	44	4	91.67 %	8.33%
Poor economic evaluation of health intervention (example: cost-benefit analysis)	39	9	81.25 %	18.75 %
Political instability/conflict	36	12	75.00 %	25.00 %
Insufficient national laws, regulations, policies, or guidelines that explicitly cover the ethical conduct of human subject research	35	13	72.92 %	27.08 %
Insufficient research that produces data to inform policy to build research capacity	32	16	66.67 %	33.33 %
Per capita cancer care expenditure	29	19	60.42 %	39.58 %
Austerity measures in the face of instability/recession	22	26	45.83 %	54.17 %
Visibility of certain types of cancers on the expense of others	19	29	39.58 %	60.42 %

Supplemental Table 6. Present political economy barriers that were selected by participants, shown as raw numbers and percentages.

Theme	Sub-theme	Number of mentions	Number of mentions (%)
Research Infrastructure	Amenities (facilities and equipment)	20	10.99%
	Research resources	12	6.59%
	Data poverty	10	5.49%
	Cancer registration	9	4.95%
Support	Financial support	32	17.58%
	Institutional support	4	2.20%
	Governmental support	3	1.65%
Logistics	Research participation	11	6.04%
	Travel	6	3.30%
	Workload and time	5	2.75%
	Collaboration	5	2.75%
	Training	3	1.65%
Human Resources		17	9.34%
Political Economy	Politics	9	4.95%
	Security	7	3.85%
Culture and Conducive Environment		15	8.24%
Others		14	7.69%

Supplemental Table 7. Themes, subthemes, and their share of mentions of research barriers from participants' responses.

Theme	Sub-theme	Number of mentions	Number of mentions (%)
Support	Financial support	21	17.21%
	Governmental support	7	5.74%
	Institutional support	3	2.46%
Logistics	Workload and time constraint	11	9.02%
	Travel and visa	8	6.56%
	Infrastructure	7	5.74%
Political Economy	Politics	13	10.66%
	Security	8	6.56%
Others		13	10.66%
Socio-Cultural	Training and research culture	8	6.56%
	Incentive to attend training	4	3.28%
Nature of Training	Quality and availability	6	4.92%
	Cost of training	5	4.10%
Human Resources		8	6.56%

Supplemental Table 8. Themes, subthemes, and their share of mentions of training barriers from participants' responses.
